# Biomarkers and outcome after tamoxifen treatment in node-positive breast cancers from elderly women

**DOI:** 10.1054/bjoc.1999.0914

**Published:** 2000-01-18

**Authors:** M G Daidone, A Luisi, G Martelli, E Benini, S Veneroni, G Tomasic, G De Palo, R Silvestrini

**Affiliations:** Oncologia Sperimentale C, Anatomia e Istologia Patologica, Semeiotica Chirurgica e Chirurgia Ambulatoriale, Istituto Nazionale per lo Studio e la Cura dei Tumori, Via Venezian 1, Milan, 20133, Italy

**Keywords:** bcl-2 expression, cell proliferation, elderly patients, hormone responsiveness, p53 expression, oestrogen receptors

## Abstract

The predictive role of tumour proliferative rate and expression of p53, bcl-2 and bax proteins, alone and in association with tumour size, nodal involvement and oestrogen receptors (ER), was analysed on 145 elderly patients (≥70 years of age) with histologically assessed node-positive breast cancers treated with radical or conservative surgery plus radiotherapy followed by adjuvant tamoxifen for at least 1 year. The 7-year probability of relapse was significantly higher for patients with tumours rapidly proliferating (hazard ratio (HR) = 2.0, *P* = 0.01), overexpressing p53 (HR = 4.4, *P* = 0.0001), weakly or not exhibiting bcl-2 (HR = 1.9, *P* = 0.02), without ERs (HR = 3.4, *P* = 0.0001) or with ≥ 4 positive lymph nodes (HR = 2.3, *P* = 0.003) than for patients with tumours expressing the opposite patho-biological profile. Conversely, tumour size and bax expression failed to influence relapse-free survival. Adjustment for the duration of tamoxifen treatment did not change these findings. Oestrogen receptors, cell proliferation, p53 accumulation and bcl-2 expression were also predictive for overall survival. Within ER-positive tumours, cell proliferation, p53 accumulation, bcl-2 expression and lymph node involvement provided significant and independent information for relapse and, in association, identified subgroups of patients with relapse probabilities of 20% (low-risk group, exhibiting only one unfavourable factor) to 90% (high-risk group, exhibiting three unfavourable factors). Such data could represent the initial framework for a biologically tailored therapy even for elderly patients and highlight the importance of a patho-biological characterization of their breast cancers. © 2000 Cancer Research Campaign


*British Journal of Cancer* (2000) **82**(2), 270–277
© 2000 Cancer Research Campaign
Article no. bjoc.1999.0914

### Biomarkers and outcome after tamoxifen treatment in node-positive breast cancers from elderly women

**MG Daidone, A Luisi, G Martelli, E Benini, S Veneroni, G Tomasic, G De Palo and R Silvestrini**

Oncologia Sperimentale C, Anatomia e Istologia Patologica, Semeiotica Chirurgica e Chirurgia Ambulatoriale, Istituto Nazionale per lo Studio e la Cura dei Tumori, Via Venezian 1, 20133 Milan, Italy

**Summary** The predictive role of tumour proliferative rate and expression of p53, bcl-2 and bax proteins, alone and in association with tumour size, nodal involvement and oestrogen receptors (ER), was analysed on 145 elderly patients (≥70 years of age) with histologically assessed node-positive breast cancers treated with radical or conservative surgery plus radiotherapy followed by adjuvant tamoxifen for at least 1 year. The 7-year probability of relapse was significantly higher for patients with tumours rapidly proliferating (hazard ratio (HR) = 2.0, $P$ = 0.01), overexpressing p53 (HR = 4.4, $P$ = 0.0001), weakly or not exhibiting bcl-2 (HR = 1.9, $P$ = 0.02), without ERs (HR = 3.4, $P$ = 0.0001) or with ≥ 4 positive lymph nodes (HR = 2.3, $P$ = 0.003) than for patients with tumours expressing the opposite patho-biological profile. Conversely, tumour size and bax expression failed to influence relapse-free survival. Adjustment for the duration of tamoxifen treatment did not change these findings. Oestrogen receptors, cell proliferation, p53 accumulation and bcl-2 expression were also predictive for overall survival. Within ER-positive tumours, cell proliferation, p53 accumulation, bcl-2 expression and lymph node involvement provided significant and independent information for relapse and, in association, identified subgroups of patients with relapse probabilities of 20% (low-risk group, exhibiting only one unfavourable factor) to 90% (high-risk group, exhibiting three unfavourable factors). Such data could represent the initial framework for a biologically tailored therapy even for elderly patients and highlight the importance of a patho-biological characterization of their breast cancers. © 2000 Cancer Research Campaign

**Keywords:** bcl-2 expression; cell proliferation; elderly patients; hormone responsiveness; p53 expression; oestrogen receptors

In the last few decades, studies on breast cancer biology have been progressively intensified, and biological characteristics have substantially contributed to improve knowledge on the natural history of the disease and to provide clinically relevant information (McGuire et al, 1992; Gasparini et al, 1993). Biological markers such as hormone and growth factor receptors, cell proliferation, DNA ploidy, genomic alterations, invasiveness markers and apoptosis-related factors have been investigated to define their prognostic role and more recently their potentials as predictors of response to local-regional or systemic treatments (Nicholson et al, 1991; Silvestrini et al, 1993*b*, 1996, 1997; Gee et al, 1994; Stal et al, 1994; Archer et al, 1995; Elledge et al, 1995*a*, 1997; Gasparini et al, 1995; Hellemans et al, 1995; Hurlimann et al, 1995; Jansson et al, 1995; Krajewski et al, 1995, 1997; Carlomagno et al, 1996; van Slooten et al, 1996; Frassoldati et al, 1997; Keen et al, 1997; Kobayashi et al, 1997; Berns et al, 1998; Clahsen et al, 1998; Paik et al, 1998; Sjøgren et al, 1998; Thor et al, 1998; Veronese et al, 1998). However, most of the biological characterizations were performed as ancillary studies in therapeutic clinical protocols which included young or middle-aged patients, and elderly women (i.e. those 65 years of age or older) were generally excluded for comorbid conditions or poor compliance with local or systemic treatments. Thus, the clinical role of biomarkers remains to be defined in tumours from elderly patients (Silliman et al, 1993), who will represent about two-thirds of those newly diagnosed each year after the year 2000 (Balducci et al, 1998).

Taking into account such an epidemiological projection, in previous reports we comparatively analysed biological profiles of tumours from young and elderly patients (Valentinis et al, 1991; Silvestrini et al, 1995*a*; Daidone et al, 1997). In the present study, considering the progressively increasing inclusion of elderly patients in clinical protocols and the evidence of treatment efficacy even in this patient population (Balducci et al, 1997), we investigated whether biological markers that are prognostic indicators in patients under 65 years of age play the same role in elderly patients. In a series of histologically assessed node-positive invasive breast cancers from patients over 70 years of age and treated with adjuvant tamoxifen, we analysed the relation between clinical outcome, oestrogen receptor (ER) and proliferative status and apoptosis-related markers.

### MATERIALS AND METHODS

### Patients and follow-up

The study comprised 145 patients over 70 years of age (median age, 74 years; range 70–88), with operable node-positive breast cancer and no clinical or radiological evidence of distant metastasis, who underwent breast-conserving surgery plus radiotherapy (46 cases) as previously described (Veronesi et al, 1981) or modified radical mastectomy (99 cases) and axillary lymph node dissection at the Milan Cancer Institute during the period February 1982 to December 1992. All patients received post-surgical adjuvant hormone therapy (tamoxifen, 20 or 30 mg $day^{-1}$) for at least 1

*Received 6 January 1999*
*Revised 22 July 1999*
*Accepted 2 August 1999*

*Correspondence to:* MG Daidone

**Table 1** Tumour characteristics

| | No. of cases | % of group |
|---|---|---|
| Size (cm) | | |
| ≤ 2 | 49 | 37 |
| > 2 | 82 | 63 |
| Unknown | 14 | |
| Positive axillary nodes | | |
| 1–3 | 70 | 48 |
| > 3 | 75 | 52 |
| ER status | | |
| Positive | 123 | 87 |
| Negative | 18 | 13 |
| Unknown | 4 | |
| TLI (%) | | |
| ≤ 3 | 71 | 49 |
| > 3 | 74 | 51 |
| $p53^+$ cells (%) | | |
| ≤ 5 | 122 | 86 |
| > 5 | 19 | 14 |
| Not assessable | 4 | |
| $bcl\text{-}2^+$ cells (%) | | |
| ≤ 30 | 75 | 55 |
| > 30 | 61 | 45 |
| Not assessable | 9 | |
| $bax^+$ cells (%) | | |
| ≤ 10 | 56 | 43 |
| > 10 | 73 | 57 |
| Not assessable | 16 | |

ER,. oestrogen receptor; TLI, [$^3$H]thymidine labelling index.

year (range 1 to more than 5 years; 67 women assuming tamoxifen for less than 2 years, and 78 for 2 years or more). Patients were consecutive for the possibility to determine the $^3$H-thymidine labelling index (TLI) on the primary tumour before starting any treatment. Tumour characteristics are reported in Table 1. Pathological tumour diameter was measurable in 131 cases; the maximum pathological diameter was under 2 cm in 37% of the cases. A similar fraction of patients had 1–3 or more than 3 positive axillary lymph nodes. The most frequent histotype was invasive ductal carcinoma, pure (95 cases) or associated with lobular (13 cases) or other histotypes (four cases).

Patients were examined at 6-month intervals during the first 5 years and at 12-month intervals thereafter. Disease status was assessed through physical examination, chest X-ray and bone scan. The median follow-up was 80 months (range 6–167 months; 25th percentile, 70; 75th percentile, 100). New disease manifestations occurred in 56 patients and were as follows: eight local-regional relapses, 47 distant metastases and one contralateral breast cancer. At 7 years from the initial diagnosis, death from any cause had occurred in 61 patients.

### In vitro determinations

Immediately after surgery, the tumour specimen was in part incubated with $^3$H-thymidine and then processed for conventional histological procedures for the determination of TLI (Silvestrini, 1991), p53 (Silvestrini et al, 1993*a*), bcl-2 (Silvestrini et al, 1994) and bax expression (Costa et al, 1998). A part of the tumour material was frozen in liquid nitrogen and stored at –80°C for the determination of ER content (Ronchi et al, 1986). The determination of proliferation index and ER was performed within national quality control programmes (Piffanelli et al, 1989; Silvestrini, 1991) recently activated also for p53 and bcl-2 expression.

### *TLI*

Small fragments of fresh tumour material was immediately incubated with $^3$H-thymidine, fixed for 1 h in Bouin solution, and processed by autoradiography using a proliferation kit (Euroframe, Asti, Italy), as previously described (Silvestrini, 1991). TLI was assessed independently by two observers by scoring a total of 1000–3000 tumour cells on different specimens from the same tumour and was defined as the percentage ratio between labelled tumour cells and total number of tumour cells.

### Immunohistochemical determinations

### *p53 expression*

Histological 4-µm sections from Bouin-fixed, paraffin-embedded blocks were incubated for 1 h with a 1:50 dilution of PAb1801 monoclonal antibody (Oncogene Science, Manhasset, NY, USA). The monoclonal antibody, which has been raised against human p53, recognizes wild-type and mutant forms of p53 protein. After incubation, the specimens were processed by using immunoperoxidase staining (Vectastain ABC Kit; Vector Laboratories, Burlingame, CA, USA) as previously described (Silvestrini et al, 1993*a*). Owing to the short time of histological fixation, microwave oven treatment was not required to obtain optimal antigen retrieval (Silvestrini et al, 1995*c*).

### *bcl-2 expression*

Histological 4-µm sections were incubated for 15 min in 0.05 M Tris–HCl (pH 7.6) containing 2% human serum albumin (Sigma) to block non-specific binding and then for 1 h at room temperature in a humidified atmosphere with a 1:40 dilution of monoclonal mouse anti-human bcl-2 oncoprotein (Dakopatts, Copenhagen, Denmark), as previously described (Silvestrini et al, 1994). After incubation, the specimens were processed by using the Dako quick-staining, labelled alkaline phosphatase kit (Dako LSAB, Dakopatts).

### *bax expression*

Histological 4-µm sections were incubated for 2 h at 4°C with a 1:400 dilution of polyclonal rabbit antibody bax N-20 (Santa Cruz Biotechnology Inc., Santa Cruz, CA, USA) as previously described (Costa et al, 1998). After incubation, the specimens were treated with a biotinylated anti-rabbit immunoglobulin and processed by using immunoperoxidase staining (Vectastain ABC Kit).

Breast cancers with high p53, bcl-2 or bax immunoreactivity were used as positive controls, whereas negative controls for the markers were obtained by omission of the primary antibody. The fraction of positive tumour cells (at a nuclear level for p53 or cytoplasmic level for bcl-2 and bax) was evaluated independently by two observers by scoring a total of 1000–3000 tumour cells and was defined as the percentage ratio between positive and total number of tumour cells.

The sensitivity of bcl-2 and bax immunohistochemical detection was confirmed by immunoblotting assays performed in a double-blind matched comparison on a limited series of breast cancer clinical specimens or cell lines (data not shown).

*© 2000 Cancer Research Campaign* 

### Oestrogen receptors

Tumour samples were immediately frozen at –25°C and stored at –80°C. Oestrogen receptor concentration was measured by the dextran-coated charcoal technique (Ronchi et al, 1986).

### Statistical analysis

For basic analysis, TLI, p53, bcl-2 and bax expression were considered as continuous variables and analysed using non-parametric approaches. The association between the fraction of TLI, p53, bcl-2 and bax expression and tumour size, lymph node involvement and ER status was assessed by means of Wilcoxon's rank-sum test. The relationship between TLI, p53, bcl-2 and bax was investigated by Spearman's regression coefficient.

When biomarkers were related to clinical outcome, they were considered as dichotomous variables by using cut-off values of prognostic relevance in large series of primary breast cancers in different clinical situations, that is 3% for TLI value (Silvestrini et al, 1995*b*, 1996); 10 fmol $mg^{-1}$ protein for ER (Di Fronzo et al, 1990); 5% of positive cells for p53 (Silvestrini et al, 1993*a*, 1996) and 30% of positive cells for bcl-2 (Silvestrini et al, 1994). For bax, a cut-off value of 10% positive cells was selected for clinical analysis according to Krajewski et al (1995), even though every tested value (from 0% to 60%) failed to identify subsets with a significantly different prognosis. Patient distribution in biological categories defined using the aforementioned criteria is reported in Table 1.

Relapse-free survival (RFS) and overall survival (OS) were computed, starting from the date of surgery, by the Kaplan–Meier product-limit method. For RFS, the occurrence of the first adverse event, in terms of local-regional relapse (i.e. local recurrence, and/or regional axillary lymph node metastasis), distant metastasis, or contralateral failure, was considered as an end point. Survival was defined as the time from surgery to death from any cause. The prognostic role of the different factors on RFS or on OS, singly or in association, was evaluated by fitting a Cox regression model. Hazard ratios (HR) and their 95% confidence limits (CL) were determined by using the putative best prognostic category as a reference.

### RESULTS

### Basic study

In the present series of elderly patients with node-positive tumours, the percentages of slowly or rapidly proliferating tumours were similar (Table 1). Sixty-two per cent of the cases showed no nuclear accumulation of the p53 protein, whereas a 1–5% fraction of p53-positive ($p53^+$) cells was detected in 24% of tumours, and >5% positive cells in 14% of tumours. Cytoplasmic bcl-2 expression was absent in 30% of cases, 1–30% of bcl-2-positive cells (bcl-2) was observed in 25% of cases, and > 30% of positive cells was detected in 45% of the cases. No cytoplasmic immunoreactivity to bax was observed in 38% of cases, 1–10% of bax-positive cells ($bax^+$) in 5% of tumours, and >10% positive cells in 57% of tumours.

TLI, bcl-2 and bax expression were generally unrelated to one another. Only p53 expression appeared to be related directly to TLI ($r_s = 0.16$, $P = 0.056$) or inversely to bcl-2 ($r_s = –0.25$, $P = 0.002$), but such relationships were very weak as shown by the low regression coefficients. Oestrogen receptors proved to be directly related to bcl-2 and bax and inversely, significantly related to p53. In fact, the fraction of $p53^+$ tumours (i.e. with more than 5% positive cells) was about five times lower in ER-positive ($ER^+$) than in ER-negative ($ER^–$) cases (9% vs 44%, $P < 0.0001$). Conversely, bcl-2 and bax expression was only weakly related to ER, since an absent or weak bcl-2 expression (i.e. less than 30% positive cells)

**Table 2** Univariate analysis of relapse-free survival at 7 years

| Variable | Unadjusted HR (95% CL) | *P*-value | HR adjusted for tamoxifen treatment duration (95% CL) | *P*-value |
|---|---|---|---|---|
| Tumour size (cm) | | | | |
| > 2 vs ≤ 2[a] | 1.3 (0.8–2.4) | 0.30 | 1.4 (0.8–2.4) | 0.28 |
| Positive nodes | | | | |
| >3 vs 1–3[a] | 2.3 (1.3–3.9) | 0.003 | 3.0 (1.7–5.2) | 0.0001 |
| ER status | | | | |
| Negative vs positive[a] | 3.4 (1.8–6.3) | 0.0001 | 2.9 (1.5–5.3) | 0.0009 |
| TLI (%) | | | | |
| > 3 vs ≤3[a] | 2.0 (1.2–3.4) | 0.01 | 1.9 (1.1–3.2) | 0.015 |
| p53+ cells (%) | | | | |
| > 5 vs ≤ 5[a] | 4.4 (2.4–8.1) | 0.0001 | 4.2 (2.3–7.6) | 0.0001 |
| bcl-2+ cells (%) | | | | |
| ≤30 vs > 30[a] | 1.9 (1.1–3.2) | 0.02 | 1.7 (1.0–2.9) | 0.06 |
| bax+ cells (%) | | | | |
| > 10 vs ≤ 10[a] | 0.8 (0.5–1.5) | 0.56 | 1.0 (0.6–1.7) | 0.9 |

[a]Reference category. ER, oestrogen receptor; TLI, [$^3$H]thymidine labelling index; HR, hazard ratio for relapse; CL, confidence limits. *P*-values referred to Wald $\chi^2$.

© 2000 Cancer Research Campaign

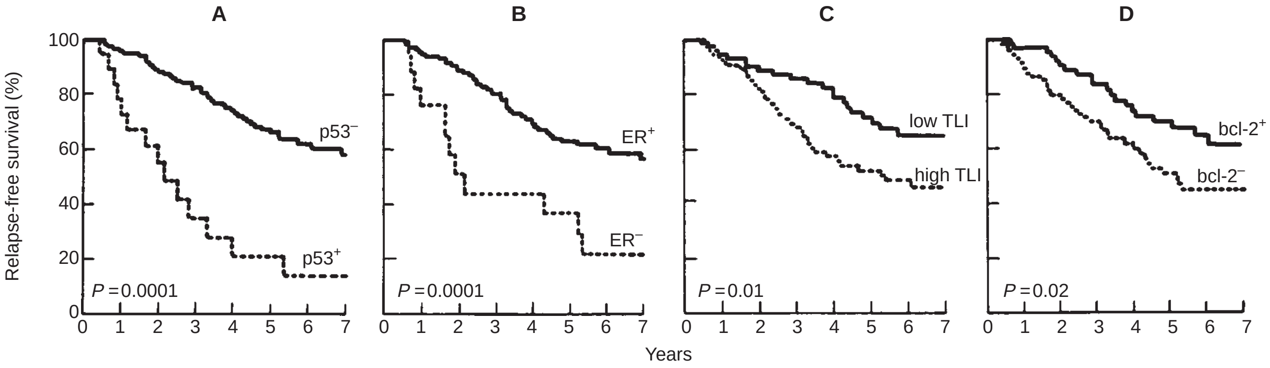

**Figure 1** Relapse-free survival curves according to p53 accumulation (**A**), ER status (**B**), TLI (**C**), and bcl-2 expression (**D**)

**Table 3** Univariate and multivariate analysis of 7-year relapse-free survival in patients with ER$^+$ tumours

| | Univariate analysis | | Multivariate analysis | |
|---|---|---|---|---|
| | HR (95% CL) | *P*-value | HR (95% CL) | *P*-value |
| Positive nodes | | | | |
| >3 vs 1–3[a] | 2.3 (1.2–4.3) | 0.01 | 2.4 (1.2–4.6) | 0.010 |
| TLI (%) | | | | |
| >3 vs ≤3[a] | 1.8 (1.0–3.4) | 0.048 | 1.9 (1.0–3.5) | 0.054 |
| p53$^+$ cells (%) | | | | |
| >5 vs ≤5[a] | 5.1 (2.3–11.3) | 0.0001 | 5.6 (2.4–13.0) | 0.0001 |
| bcl-2$^+$ cells (%) | | | | |
| ≤ 30 vs >30[a] | 2.0 (1.1–3.7) | 0.03 | 2.0 (1.1–3.8) | 0.025 |

[a]Reference category. ER, oestrogen receptor; TLI, [$^3$H]thymidine labelling index; HR, hazard ratio for relapse; CL, confidence limits. *P*-values referred to Wald $\chi^2$.

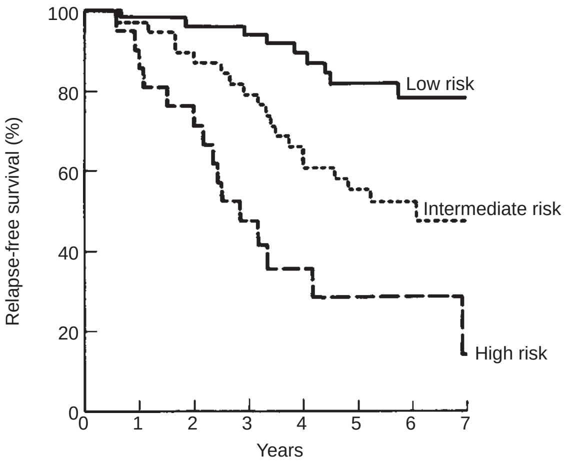

**Figure 2** Relapse-free survival curves in the subset of patients with ER$^+$ tumours as a function of the risk profile defined by p53 accumulation, TLI, bcl-2 expression and lymph node involvement. Low risk, presence of only one unfavourable factor (>3 positive axillary lymph nodes, TLI >3%, p53-positive cells >5%, or bcl-2-positive cells ≤30%). Intermediate risk, presence of two unfavourable factors. High risk, presence of three unfavourable factors was more frequent in ER$^-$ than in ER$^+$ cases (72% vs 52%, $P$ = 0.10), as was an absent or weak bax expression (i.e. less than 10% positive cells), which was present in 40% of ER$^+$ and in 67% of ER$^-$ tumours ($P$ = 0.07). No relationship was observed between TLI, ER, p53, bcl-2 or bax expression and pathological variables, i.e. tumour size and nodal involvement.

### Clinical outcome as a function of biological variables

Univariate analysis showed that p53 expression, ER status, TLI and bcl-2 expression, together with lymph node involvement, were significant predictors of RFS within 7 years of surgery, whereas bax expression and tumour size did not influence RFS (Table 2). Such results, with minimal changes in the HR (Table 2), held true even after adjustment for the duration of tamoxifen treatment, which singly also significantly influenced clinical outcome (< 2 years vs ≥ 2 years: HR for relapse = 1.7, 95% CL = 1.0–2.8, $P$ = 0.037; HR for death = 2.4, 95% CL = 1.4–4.0, $P$ = 0.0001). RFS curves as a function of p53 appeared divergent starting from the first year of follow-up, and such a difference markedly increased with time (Figure 1A). At 7 years from surgery, only five of the 19 patients with p53$^+$ tumours were still relapse-free compared to 79 of the 122 women with p53$^+$ cancers. RFS curves as a function of ER also appeared diversified starting from the first year of follow-up (Figure 1B), whereas for TLI and bcl-2 expression curves diversified at a longer time (Figure 1C,D). Consideration of bax expression did not provide predictive information even when analysed in association with p53 accumulation, bcl-2 expression, TLI or ER status.

Even within the more substantial subset of ER$^+$ tumours (123 cases), besides nodal involvement, TLI, bcl-2 expression and p53 accumulation also maintained their predictive relevance in univariate analysis (Table 3), whereas tumour size (> 2 vs ≤ 2 cm, HR = 1.2, 95% CL = 0.6–2.2, $P$ = 0.64) and bax expression (positive vs negative, HR = 0.9, 95% CL = 0.5–1.6, $P$ = 0.63) still had no influence on RFS. The predictive information provided by patho-biological variables was maintained in a multivariate

© 2000 Cancer Research Campaign

**Table 4** Univariate analysis of overall survival at 7 years

| Variable | Overall series % | HR (95% CL) | *P*-value | ER+ tumours % | HR (95% CL) | *P*-value |
|---|---|---|---|---|---|---|
| Tumour size (cm) | | | | | | |
| ≤ 2[a] | 61 | – | | 62 | – | |
| > 2 | 53 | 1.4 (0.8–2.4) | 0.29 | 59 | 1.2 (0.6–2.3) | 0.53 |
| Positive nodes | | | | | | |
| 1–3[a] | 61 | – | | 62 | – | |
| >3 | 53 | 1.4 (0.8–2.4) | 0.18 | 58 | 1.2 (0.7–2.2) | 0.49 |
| ER status | | | | | | |
| Positive[a] | 60 | – | | | | |
| Negative | 33 | 2.7 (1.4–5.1) | 0.0022 | | | |
| TLI (%) | | | | | | |
| ≤ 3[a] | 65 | – | | 69 | – | |
| > 3 | 49 | 1.7 (1.0–2.8) | 0.05 | 53 | 1.6 (1.0–2.9) | 0.08 |
| p53+ cells (%) | | | | | | |
| ≤5[a] | 61 | – | | 64 | – | |
| >5 | 26 | 3.8 (2.1–7.0) | 0.0001 | 18 | 5.8 (2.7–12.2) | 0.0001 |
| bcl-2+ cells (%) | | | | | | |
| > 30[a] | 63 | – | | 68 | – | |
| ≤ 30 | 49 | 1.6 (1.0–2.8) | 0.07 | 50 | 1.9 (1.0–3.4) | 0.04 |
| bax+ cells (%) | | | | | | |
| ≤ 10[a] | 57 | – | | 61 | – | |
| > 10 | 55 | 1.0 (0.6–1.7) | 0.9 | 58 | 1.1 (0.6–2.1) | 0.74 |

[a]Reference category. ER, oestrogen receptor; TLI, [$^3$H]thymidine labelling index; HR, hazard ratio for death; CL, confidence limits. *P*-values referred to Wald $\chi^2$.

analysis carried out on the 117 $ER^+$ tumours for which all the biologic data were available (Table 3). In this subset of patients, biological and pathological information was considered together in a descriptive analysis to investigate whether a multivariate characterization could improve the predictivity on relapse of each variable, singly considered. Using the regression model estimates, three subgroups of patients with different RFS probabilities were identified (Figure 2): a low-risk group, with a 20% probability of relapse (55 cases, nine events), characterized by no or weak p53 expression and by the presence of only one unfavourable factor (high TLI, weak-absent bcl-2 expression, or more than three positive axillary lymph nodes); a high-risk group, with a probability of relapse of about 90% (22 cases, 15 events), characterized by the presence of three unfavourable factors among those previously mentioned, also including p53 overexpression; and an intermediate-risk group, with a relapse probability of about 50% (40 cases, 19 events), in which two unfavourable factors were present. The HRs for relapse of the last two subsets were significantly different from that of the favourable subset (intermediate-risk vs low-risk subset, HR = 3.2, 95% CL = 1.4–7.0, $P = 0.0043$; high-risk vs low-risk subset, HR = 8.2, 95% CL = 3.5–18.8, $P = 0.0001$).

On the overall series of 145 cases, ER status, p53 accumulation, TLI and bcl-2 expression (but not tumour size, nodal involvement or bax expression) singly predicted 7-year survival after surgery and tamoxifen therapy (Table 4). These findings held true after adjustment for the duration of tamoxifen treatment (data not shown) and within the subset of 123 patients with $ER^+$ tumours (Table 4).

### DISCUSSION

In the past, the interest of clinicians in planning innovative therapeutic protocols has rarely focused on breast cancer patients over 70 years of age. Such an attitude was also due to the belief that elderly patients generally develop indolent disease and are less responsive to treatment. However, several studies have demonstrated that adjuvant therapy with tamoxifen is able to improve RFS and OS in patients older than 70 years. Such a benefit has been mainly observed in patients with $ER^+$ tumours and, although to a lesser degree, also in those with $ER^-$ tumours (Martelli et al, 1995; Early Breast Cancer Trialists' Collaborative Group, 1998). Therefore, there is renewed scientific interest in the biological characterization of tumours from elderly women.

In the present study, the influence of several biological factors – which are associated with or indicative of different cellular functions and which are only weakly related to one another – on clinical outcome was investigated in a subset of node-positive patients over 70 years old who underwent radical or conservative surgery with axillary lymph node dissection followed by tamoxifen treatment. Besides lymph node involvement, cell proliferation, p53 and bcl-2 expression and ER status were predictors of overall relapse, whereas tumour size and bax expression did not provide predictive information on RFS. Such findings held true even for OS and after adjustment for the duration of tamoxifen treatment. Lymph node involvement, cell proliferation, and p53 and bcl-2 expression were independent prognostic discriminants for relapse even within the

 © 2000 Cancer Research Campaign subset of patients with ER+ tumours, i.e. in women traditionally considered responsive to endocrine treatment and who markedly benefit from adjuvant tamoxifen. In addition, within ER+ tumours, the favourable biological profile (characterized by the absence of or a weak p53 accumulation) was further defined by the presence of other favourable factors, such as slow proliferation, bcl-2 overexpression, and less than 3 involved axillary lymph nodes. All the features, in association, identified patients with a high probability to be relapse free at 7 years after starting tamoxifen treatment (who accounted for about 50% of the cases). Conversely, an aggressive patho-biological profile, characterized by the presence of two or three of the four unfavourable factors (among which also p53 overexpression was included), identified patients who partially (in 50% of the cases, accounting for about 30% of the subset) or totally (in 90% of cases, accounting for about 20% of the subset) escaped tamoxifen control notwithstanding the presence of ER and who therefore were candidate for different types of treatment. Although the analysis is exploratory and data are hypothesis generating and need to be validated by retrospective or prospective studies on independent series of patients, the latter could represent a preliminary framework for a biologically tailored therapy even for elderly breast cancer patients.

The role of cell proliferation, p53 accumulation, bcl-2 and bax expression, in addition to lymph node involvement and ER status, in predicting clinical outcome following endocrine treatment has been investigated by several authors in adjuvant or advanced settings of younger patients (Paradiso et al, 1990; Nicholson et al, 1991; Silvestrini et al, 1993*b*, 1996; Gee et al, 1994; Archer et al, 1995; Gasparini et al, 1995; Hellemans et al, 1995; Hurlimann et al, 1995; Elledge et al, 1997; Kobayashi et al, 1997; Berns et al, 1998; Veronese et al, 1998). Overall, the outcome of the retrospective studies has been confirmed in our experience on elderly patients. In fact, low proliferative rate (Paradiso et al, 1990; Nicholson et al, 1991; Silvestrini et al, 1993*b*, 1996; Archer et al, 1995) and absence of p53 alterations – determined by using several different approaches (Gasparini et al, 1995; Silvestrini et al, 1996; Elledge et al, 1997; Berns et al, 1998), and bcl-2 overexpression (Gee et al, 1994; Gasparini et al, 1995; Hellemans et al, 1995; Silvestrini et al, 1996; Elledge et al, 1997; Kobayashi et al, 1997; Veronese et al, 1998) appeared as indicators of a favourable outcome following endocrine treatment in patients with limited disease and, in some instances, even as predictors of treatment response in patients with advanced disease.

In our experience (Silvestrini et al, 1995*a*, 1996; Daidone et al, 1997), ER tumours of elderly patients differed of those from younger patients also for a lower frequency of features indicative of biological aggressiveness, such as rapid proliferation (detected in 51% vs 60% of the cases) or p53+ overexpression (detected in 9% vs 15% of the cases), and for a higher frequency of bcl-2-expressing cells (52% vs 21%). The higher frequency of bcl-2-expressing tumours can be explained by the prevalence of well- and moderately differentiated tumours in elderly than in younger patients (Hyman and Muss, 1994). Such evidence could support the hypothesis that, in cancers from epithelial origin, bcl-2 expression may allow differentiation, thereby preventing programmed cell death (Nathan et al, 1994) and could also explain the valuable clinical role of bcl-2 in elderly patients. An absent or weak bcl-2 expression is by itself an unfavourable prognostic marker of the natural history of node-negative breast cancer treated with local-regional therapy (Silvestrini et al, 1994) and remains singly an unfavourable factor in node-positive and advanced tumours from pre- and post-menopausal patients given endocrine therapy (Gee et al, 1994; Gasparini et al, 1995; Hellemans et al, 1995; Silvestrini et al, 1996; Elledge et al, 1997; Keen et al, 1997; Kobayashi et al, 1997; Veronese et al, 1998). Our findings provide further insight about the hypothesis of bcl-2 expression as a differentiation marker or a surrogate marker for other molecular or biological processes related to hormone sensitivity rather than a predictor of response to hormonal treatment (Elledge et al, 1997). In fact, its association with clinical outcome following endocrine treatment could be due to an identification of indolent, well-differentiated tumours rather than to a direct involvement of the anti-apoptotic marker in determining sensitivity to tamoxifen treatment. Our finding that bax expression does not improve the clinical predictivity of bcl-2 alone, in agreement with a previous report (Veronese et al, 1998), is in keeping with such a hypothesis.

p53 accumulation and proliferative rate confirmed even in elderly patients their important role as predictors of clinical outcome following hormonal treatment, as already emerged in younger patients (Nicholson et al, 1991; Archer et al, 1995; Gasparini et al, 1995; Silvestrini et al, 1996; Berns et al, 1998; Elledge et al, 1997). In particular, p53 appeared as a much stronger determinant of RFS in elderly than in younger patients, since its overexpression, although present in a limited fraction of cases, is associated to an 85% probability of relapse. However, based on preclinical (Elledge et al, 1995*b*) and clinical studies on advanced tumours (Elledge et al, 1997), its predictivity on clinical outcome following tamoxifen should not indicate an actual role as a predictor of tumour sensitivity to hormonal treatment. Our findings are in keeping with the hypothesis that alterations in p53 expression are associated to a more aggressive and undifferentiated phenotype and for this reason can provide additive information to conventional predictors of tumour response to endocrine therapy, such as ER.

The present results are in favour of a similarity in the clinical role of biological markers in tumours from young and elderly women. Although the data need to be confirmed on independent data sets, they further stress the importance of a biological characterization even on tumours from elderly patients. The identification of different patho-biological phenotypes, besides improving the prognostic and predictive armamentarium, could help to design treatments with a favourable cost/benefit balance for patients. It will represent an important target for health services in the future, since such tumours in the elderly will account for about two-thirds of all breast cancers and for 70% of breast cancer deaths by the year 2010 (Balducci et al, 1998).

### ACKNOWLEDGEMENTS

Supported by grants from the Ministero della Sanità, the Associazione Italiana per la Ricerca sul Cancro, and Consiglio Nazionale delle Ricerche. We thank B Canova for preparing the manuscript, B Johnston for editorial assistance, and L Dressino for his skilled technical assistance.

### REFERENCES

Archer SG, Eliopoulos A, Spandidos D, Barnes D, Ellis IO, Blamey RW, Nicholson RI and Robertson JFR (1995) Expression of ras p21, p53 and c-erbB-2 in advanced breast cancer and response to first line hormonal therapy. *Br J Cancer* **72**: 1259–1266

Balducci L, Extermann M, Fentiman I, Monfardini S and Perrone F (1997) Should adjuvant chemotherapy be used to treat breast cancer in elderly patients (≥70 years of age)? *Eur J Cancer* **33**: 1720–1724

Balducci L, Silliman RA and Baekey P (1998) Breast cancer: an oncological perspective – part 1. In: *Comprehensive Geriatric Oncology*, Balducci L, Lyman GH and Ershler WB (eds), pp. 629–660. Harwood Academic Publishers: Amsterdam.

Berns EMJJ, Klijn JGM, van Putten WLJ, de Witte HH, Look MP, Meijer-van Gelder ME, Willman K, Portengen H, Benraad TJ and Foekens JA (1998) p53 protein accumulation predicts poor response to tamoxifen therapy of patients with recurrent breast cancer. *J Clin Oncol* **16**: 121–127

Carlomagno C, Perrone F, Gallo C, De Laurentiis M, Lauria R, Morabito A, Pettinato G, Panico L, D'Antonio A, Bianco AR and De Placido S (1996) c-erbB2 overexpression decreases the benefit of adjuvant tamoxifen in early-stage breast cancer without axillary lymph node metastases. *J Clin Oncol* **14**: 2702–2708

Clahsen PC, van de Velde CJH, Duval C, Pallud C, Mandard A-M, Delobelle-Deroide A, van den Broek L, Sahmoud TM and van de Vijver MJ (1998) p53 protein accumulation and response to adjuvant chemotherapy in premenopausal women with node-negative early breast cancer. *J Clin Oncol* **16**: 470–479

Costa A, Licitra L, Veneroni S, Daidone MG, Grandi C, Cavina R, Molinari R and Silvestrini R (1998) Biological markers as indicators of pathological response to primary chemotherapy in oral cavity cancers. *Int J Cancer (Pred Oncol)* **79**: 613–623

Daidone MG, Luisi A, Di Fronzo G and Silvestrini R (1997) Biological characteristics of primary breast cancer in elderly. In: *Comprehensive Geriatric Oncology*, Balducci L, Lyman GH, Cuervo Henderson C and Ershler WB (eds), pp. 197–200. Harwood Academic: Amsterdam

Di Fronzo G, Coradini D, Cappelletti V, Miodini P, Granata G, Schwartz M and Panko WB (1990) Hormone receptors and disease-free survival in breast cancer: impact of increasing threshold levels. *Anticancer Res* **10**: 1699–1706

Early Breast Cancer Trialists' Collaborative Group (1998) Tamoxifen for early breast cancer: an overview of the randomised trials. *Lancet* **351**: 1451–1467

Elledge RM, Gray R, Mansour E, Yu Y, Clark GM, Ravdin P, Osborne CK, Gilchrist K, Davidson NE, Robert N, Tormey DC and Allred DC (1995*a*) Accumulation of p53 protein as a possible predictor of response to adjuvant combination chemotherapy with cyclophosphamide, methotrexate, fluorouracil, and prednisone for breast cancer. *J Natl Cancer Inst* **87**: 1254–1256

Elledge RM, Lock-Lim S, Allred D, Hilsenbeck SG and Cordner L (1995*b*) p53 mutation and tamoxifen resistance in breast cancer. *Clin Cancer Res* **1**: 1203–1208

Elledge RM, Green S, Howes L, Clark GM, Berardo M, Allred DC, Pugh R, Ciocca D, Ravdin P, O'Sullivan J, Rivkin S, Martino S and Osborne CK (1997) bcl-2, p53, and response to tamoxifen in estrogen receptor-positive metastatic breast cancer: a Southwest Oncology Group Study. *J Clin Oncol* **15**: 1916–1922

Frassoldati A, Adami F, Banzi C, Criscuolo M, Piccinini L and Silingardi V (1997) Changes of biological features in breast cancer cells determined by primary chemotherapy. *Breast Cancer Res Treat* **44**: 185–192

Gasparini G, Pozza F and Harris AL (1993) Evaluating the potential usefulness of new prognostic and predictive indicators in node-negative breast cancer patients. *J Natl Cancer Inst* **85**: 1206–1219

Gasparini G, Barbareschi M, Doglioni C, Dalla Palma P, Mauri FA, Boracchi P, Bevilacqua P, Caffo O, Morelli L, Verderio P, Pezzella F and Harris AL (1995) Expression of bcl-2 protein predicts efficacy of adjuvant treatments in operable node-positive breast cancer. *Clin Cancer Res* **1**: 189–198

Gee JM, Robertson JFR, Ellis IO, Willsher P, McClelland RA, Hoyle HB, Kyme SR, Finlay P, Blamey RW and Nicholson RI (1994) Immunocytochemical localization of bcl-2 protein in human breast cancers and its relationship to a series of prognostic markers and response to endocrine therapy. *Int J Cancer* **59**: 619–628

Hellemans P, Van Dam PA, Weyler J, Van Oosterom AT, Buytaert P and Van Marck E (1995) Prognostic value of bcl-2 expression in invasive breast cancer. *Br J Cancer* **72**: 354–360

Hurlimann J, Larrinaga B and Vala DLM (1995) bcl-2 protein in invasive ductal breast carcinomas. *Vichows Archiv* **426**: 163–168

Hyman B and Muss MD (1994) The role of chemotherapy and adjuvant therapy in the management of breast cancer in older women. *Cancer* **74**: 165–171

Jansson T, Inganas M, Sjogren S, Norberg T, Lindgren A, Holmberg L and Bergh J (1995) p53 status predicts survival in breast cancer patients treated with or without postoperative radiotherapy: a novel hypothesis based on clinical findings. *J Clin Oncol* **13**: 2745–2751

Keen JC, Dixon JM, Miller EP, Cameron DA, Chetty U, Hanby A, Bellamy C and Miller WR (1997) The expression of Ki-S1 and bcl-2 and the response to primary tamoxifen therapy in elderly patients with breast cancer. *Breast Cancer Res Treat* **44**: 123–133

Kobayashi S, Iwase H, Ito Y, Yamashita H, Iwata H, Yamashita T, Ito K, Toyama T, Nakamura T and Masaoka A (1997) Clinical significance of bcl-2 gene expression in human breast cancer tissues. *Br Cancer Res Treat* **42**: 173–181

Krajewski S, Blomqvist C, Franssila K, Krajewska M, Wasenius VM, Niskanen E, Nordling S and Reed JC (1995) Reduced expression of proapoptotic gene bax is associated with poor response rate to combination chemotherapy and shorter survival in women with metastatic breast adenocarcinoma. *Cancer Res* **55**: 4471–4478

Krajewski S, Thor AD, Edgerton SM, Moore II DH, Krajewska M and Reed JC (1997) Analysis of bax and bcl-2 expression in p53-immunopositive breast cancers. *Clin Cancer Res* **3**: 199–208

Martelli G, De Palo G, Rossi N, Coradini D, Boracchi P, Galante E and Vetrella G (1995) Long-term follow-up of elderly patients with operable breast cancer treated with surgery without axillary dissection plus adjuvant tamoxifen. *Br J Cancer* **72**: 1252–1255

McGuire WL, Tandon AK, Allred DG, Chamness GC, Ravdin PN and Clark GM (1992) Prognosis and treatment decisions in patients with breast cancer without axillary node involvement. *Cancer* **70**: 1775–1781

Nathan B, Gusterson B, Jadayel D, O'Hare M, Anbazhagan R, Jayatilake H, Ebbs S, Micklem K, Price K, Gelber R, Reed R, Senn HJ, Gold Hirscha and Dyer MJS (1994) Expression of bcl-2 in primary breast cancer and its correlation with tumour phenotype. *Ann Oncol* **5**: 409–414

Nicholson RI, Bouzubar N, Walker KJ, McClelland R, Dixon AR, Robertson JF, Ellis IO and Blamey RW (1991) Hormone sensitivity in breast cancer: influence of heterogeneity of oestrogen receptor expression and cell proliferation. *Eur J Cancer* **27**: 908–913

Paik S, Bryant J, Park C, Fisher B, Tan-Chiu E, Hyams D, Fisher ER, Lippman ME, Wickerham DL and Wolmark N (1998) ErbB-2 and response to doxorubicin in patients with axillary lymph node-positive, hormone receptor-negative breast cancer. *J Natl Cancer Inst* **90**: 1361–1370

Paradiso A, Tommasi S, Mangia A, Simone G and De Lena M (1990) Tumor proliferative activity, progesterone receptor status, estrogen receptor level, and clinical outcome of estrogen receptor-positive advanced breast cancer. *Cancer Res* **50**: 2958–2962

Piffanelli A, Pelizzola D, Giovannini G, Catozzi L, Faggioli L and Giganti M (1989) Characterization of laboratory working standards for quality control of immunometric and radiometric estrogen receptor assays. Clinical evaluation on breast cancer biopsies. *Tumori* **75**: 550–556

Ronchi E, Granata G, Brivio M, Coradini D, Miodini P and Di Fronzo G (1986) A double-labeling assay for simulataneous estimation and characterization of estrogen and progesterone receptors using radioiodinated estradiol and tritiated org 2058. *Tumori* **72**: 251–257

Silliman RA, Balducci L, Goodwin JS, Holmes FF and Leventhal EA (1993) Breast cancer care in old age: what we know, don't know, and do. *J Natl Cancer Inst* **85**: 190–199

Silvestrini R (on behalf of the SICCAB Group for Quality Control of Cell Kinetic Determination) (1991) Feasibility and reproducibility of $^3$H-thymidine labeling index in breast cancer. *Cell Prolif* **24**: 437–445

Silvestrini R, Benini E, Daidone MG, Veneroni S, Boracchi P, Cappelletti V, Di Fronzo G and Veronesi U (1993*a*) p53 as an independent prognostic marker in lymph node-negative breast cancer patients. *J Natl Cancer Inst* **85**: 965–970

Silvestrini R, Daidone MG, Mastore M, Di Fronzo G, Coradini D, Boracchi P, Squicciarini P, Salvadori B and Veronesi U (1993*b*) Cell kinetics as a predictive factor in node-positive breast cancer treated with adjuvant hormone therapy. *J Clin Oncol* **11**: 1150–1155

Silvestrini R, Veneroni S, Daidone MG, Benini E, Boracchi P, Mezzetti M, Di Fronzo G, Rilke F and Veronesi U (1994) The bcl-2 protein: a prognostic indicator strongly related to p53 protein in lymph node-negative breast cancer patients. *J Natl Cancer Inst* **86**: 499–504

Silvestrini R, Benini E, Luisi A, Veneroni S, Valentinis B and Daidone MG (1995*a*) Biology of breast cancer in elderly women. In: *Mastology – Breast Disease*. ASS Figueira Fo (ed), pp. 255–258

Silvestrini R, Daidone MG, Luisi A, Boracchi P, Mezzetti M, Di Fronzo G, Andreola S, Salvadori B, Veronesi U (1995*b*) Biological and clinicopathological factors as indicators of specific relapse types in node-negative breast cancer. *J Clin Oncol* **13**: 697–704

Silvestrini R, Rao S, Benini E, Daidone MG and Pilotti S (1995*c*) Immunohistochemical detection of p53 in clinical breast cancers: a look at methodologic approaches (Letter). *J Natl Cancer Inst* **87**: 1020

Silvestrini R, Benini E, Veneroni S, Daidone MG, Tomasic G, Squicciarini P, Salvadori B (1996) p53 and bcl-2 expression correlates with clinical outcome in a series of node-positive breast cancer patients. *J Clin Oncol* **14**: 1604–1610

 *© 2000 Cancer Research Campaign*

Silvestrini R, Veneroni S, Benini E, Daidone MG, Luisi A, Leutner M, Maucione A, Kenda R, Zucali R and Veronesi U (1997) Expression of p53, GST-π and bcl-2 and benefit from adjuvant radiotherapy in breast cancer. *J Natl Cancer Inst* **89**: 639–645

Sjøgren S, Inganas M, Lindgren A, Holmberg L and Bergh J (1998) Prognostic and predictive value of c-erbB-2 overexpression in primary breast cancer, alone and in combination with other prognostic markers. *J Clin Oncol* **16**: 462–469

Stal O, Skoog L, Rutqvist LE, Carstensen JM, Wingren S, Sullivan S, Andersson AC, Dufmats M and Nordenskjold B (1994) S-phase fraction and survival benefit from adjuvant chemotherapy or radiotherapy of breast cancer. *Br J Cancer* **70**: 1258–1262

Thor AD, Berry DA, Budman DR, Muss HB, Kute T, Henderson IC, Barcos M, Cirrincione C, Edgerton S, Allred C, Norton L and Liu ET (1998) ErbB-2, p53, and efficacy of adjuvant therapy in lymph node-positive breast cancer. *J Natl Cancer Inst* **90**: 1346–1360

Valentinis B, Silvestrini R, Daidone MG, Coradini D, Galante E, Cerrotta AM, Abolafio G and Arboit L (1991) $^{3}$H-Thymidine labeling index, hormone receptors and ploidy in breast cancer from elderly patients. *Breast Cancer Res Treat* **20**: 19–24

van Slooten HJ, Clahsen PC, van Dierendonck JH, Duval C, Pallud C, Mandard AM. Delobelle-Deroide A, van de Velde CJ and van de Vijver MJ (1996) Expression of bcl-2 in node-negative breast cancer is associated with various prognostic factors, but does not predict response to one course of perioperative chemotherapy. *Br J Cancer* **74**: 78–85

Veronese S, Mauri FA, Caffo O, Scaioli M, Aldovini D, Perrone G, Galligioni E, Doglioni C, Dalla Palma P and Barbareschi M (1998) Bax immunohistochemical expression in breast carcinoma: a study with long term follow-up. *Int J Cancer (Pred Oncol)* **79**: 13–18

Veronesi U, Saccozzi R, Del Vecchio M, Banfi A, Clemente C, De Lena M, Gallus G, Greco M, Luini A, Marubini E, Muscolino G, Rilke F, Salvadori B, Zecchini A and Zucali R (1981) Comparing radical mastectomy with quadrantectomy, axillary dissection, and radiotherapy in patients with small cancers of the breast. *N Engl J Med* **305**: 6–11

© 2000 Cancer Research Campaign
